# A retrospective comparative study on the diagnostic efficacy and the complications: between CassiII rotational core biopsy and core needle biopsy

**DOI:** 10.3389/fonc.2023.1067246

**Published:** 2023-09-26

**Authors:** Shuduo Xie, Siwei Ju, Xun Zhang, Chao Qi, Jiahang Zhang, Misha Mao, Cong Chen, Yongxia Chen, Feiyang Ji, Jichun Zhou, Linbo Wang

**Affiliations:** ^1^ Department of Surgical Oncology, Affiliated Sir Run Run Shaw Hospital, Zhejiang University School of Medicine, Hangzhou, Zhejiang, China; ^2^ Key Laboratory of Cancer Prevention and Intervention, Ministry of Education, Hangzhou, Zhejiang, China; ^3^ Department of Clinical Laboratory, Affiliated Sir Run Run Shaw Hospital, Zhejiang University School of Medicine, Hangzhou, Zhejiang, China; ^4^ Department of Breast Surgery, Affiliated Hangzhou First People’s Hospital, Zhejiang University School of Medicine, Hangzhou, Zhejiang, China; ^5^ Zhejiang Provincial Clinical Research Center for CANCER, Hangzhou, Zhejiang, China

**Keywords:** CassiII rotational core biopsy, core needle biopsy, breast cancer, carcinoma in situ, invasive breast cancer, hormonal receptor, HER2, Ki 67

## Abstract

Accurate pathologic diagnosis and molecular classification of breast mass biopsy tissue is important for determining individualized therapy for (neo)adjuvant systemic therapies for invasive breast cancer. The CassiII rotational core biopsy system is a novel biopsy technique with a guide needle and a “stick-freeze” technology. The comprehensive assessments including the concordance rates of diagnosis and biomarker status between CassiII and core needle biopsy were evaluated in this study. Estrogen receptor (ER), progesterone receptor (PgR), human epidermal growth factor receptor 2 (HER2), and Ki67 were analyzed through immunohistochemistry. In total, 655 patients with breast cancer who underwent surgery after biopsy at Sir Run Run Shaw Hospital between January 2019 to December 2021 were evaluated. The concordance rates (CRs) of malignant surgical specimens with CassiII needle biopsy was significantly high compared with core needle biopsy. Moreover, CassiII needle biopsy had about 20% improvement in sensitivity and about 5% improvement in positive predictive value compared to Core needle biopsy. The characteristics including age and tumor size were identified the risk factors for pathological inconsistencies with core needle biopsies. However, CassiII needle biopsy was associated with tumor diameter only. The CRs of ER, PgR, HER2, and Ki67 using Cassi needle were 98.08% (kappa, 0.941; p<.001), 90.77% (kappa, 0.812; p<.001), 69.62% (kappa, 0.482; p<.001), and 86.92% (kappa, 0.552; p<.001), respectively. Post-biopsy complications with CassiII needle biopsy were also collected. The complications of CassiII needle biopsy including chest stuffiness, pain and subcutaneous ecchymosis are not rare. The underlying mechanism of subcutaneous congestion or hematoma after CassiII needle biopsy might be the larger needle diameter and the effect of temperature on coagulation function. In summary, CassiII needle biopsy is age-independent and has a better accuracy than CNB for distinguishing carcinoma *in situ* and invasive carcinoma.

## 1 Introduction

Breast cancer (BC) has become the most commonly diagnosed cancer worldwide and the leading cause of cancer death among females (1). Malignant breast tumors, as a heterogeneous disease, which is classified into at least four distinct phenotypes demonstrate diverse clinical behaviors and therapeutic responsiveness (2, 3). Immunohistochemistry for hormonal receptors [HR, including estrogen receptor (ER) and progesterone receptor (PgR)], human epidermal growth factor receptor 2 (HER2) and Ki67-index (Ki67) can be used for classifying the approximate molecular subtype and deciding the most appropriate and effective therapies for individuals.

Tissue biopsy, as a way of preoperative pathological diagnosis of potentially malignant breast lesions, has the advantage of guiding the selection of surgical methods or scheduling the neoadjuvant therapies for malignancy patients. Fine-needle aspiration biopsy (FNAB) was first introduced in 1930 by Martin and Ellis and became popular in Europe (4). However, the utility of FNAB is on the decline as the consequence of its diagnostic accuracy limitation, decreased discernibility between *in situ* versus invasive cancer and has been replaced with core needle biopsy (CNB) (5–7). CNB is a diagnostic procedure where a radiologist or surgeon utilizes a hollow needle (usually 8- to 16-gauge) to aspirate 3 to 5 cores of tissue from the suspicious area in the breast. CNB which was introduced in late 1990s provides with high accuracy and more tissue for grading tumors (8). CNB makes it possible to assess predictive factors like ER, PgR, HER2 and Ki67 (6). It has been reported that the sensitivity and specificity values range from 85%–100% and 86%–100% for CNB and the concordance rates (CRs) of biomarker status between needle biopsy and surgical excision (SE) are high but variable, from 79%-100% (9, 10). It is generally believed that CNB should take more than 4 specimens for histological examination. Therefore, the disadvantages are that it requires multiple injections and makes it difficult to puncture small masses due to the ejection effect of CNB (11, 12). In 1995, Vacuum assisted biopsy (VAB) of the breast was originally developed by a radiologist Fred Burbank and Mark Retchard with the aim to improve the deficiencies of the automatic core biopsy gun technique (13). The VAB device sucks tissue into the aperture of the needle which is then sliced off with the rotating cutter. The specimen is then transported to a port chamber usually without the need to remove and re-insert the needle. It can be very appropriately used to resect breast lesions with a therapeutic intent. The appropriate application of VAB in the management of breast diseases is still controversial and VAB is mainly used in the excision of benign breast tumors rather than the preferred method of biopsy in China (14, 15).

Since the rollout of the CassiII Rotational Core Biopsy device (referred to as CassiII afterwards) is from 2019, it is a new biopsy technique with a 19-gauge guide needle to locate the biopsy site and the “stick-freeze” technology to secure the targeted tissue to the needle (16). The 10- or 12-gauge cutting cannula then rotates over the needle to provide a cylindrical and large tissue sample. It is performed by a surgeon to obtain larger and more complete specimens with less operating frequency. At the same time, surgeons can receive the preliminary report of frozen sections quickly within 30 minutes after biopsy, which provides the possibility of rapid diagnosis of disease. The accuracy values for CassiII needle biopsy and the CRs of biomarker status between CassiII biopsy and CNB have not been reported yet. The potential post-operation complications have also not been reported.

In this study, we evaluated the pathological consistency with CassiII between biopsy and surgical specimen and compared the accuracy and consistency in breast cancer between two needle biopsies including CassiII and CNB. We also aimed to evaluate and analyze the accuracy and the CRs of hormonal receptors, HER2 and Ki67 in potentially malignant breast lesions patients between biopsy tissue and surgical specimens either with CassiII or CNB. We also collected complications after CassiII needle biopsy and evaluated the effects of temperature on activated Partial Thromboplastin Time (APTT) to investigate the underlying mechanism of not rare subcutaneous ecchymosis after CassiII needle biopsy.

## 2 Materials and methods

### 2.1 Patients and tissues samples

**Figure 1:**
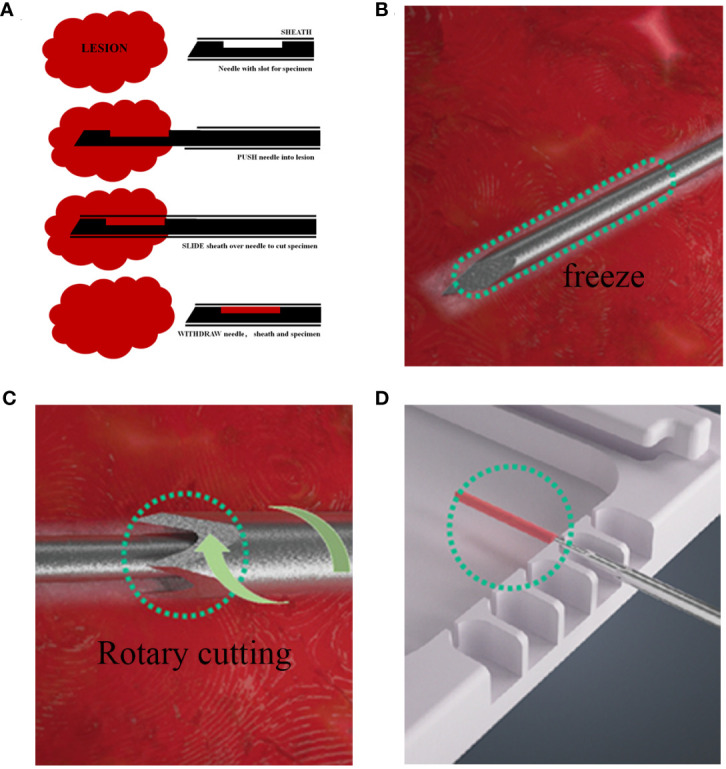
Different steps in the CNB and CassiII biopsy procedure. **(A)** Procedure for CNB biopsy. **(B)** The Cassi guided needle is guided under the image into the lesion, followed by CO_2_ flow to the tip of the guided needle, where heat exchange occurs, causing the tip to cool down and freeze the lesion. **(C)** The cutting cannula then rotates over the needle to provide a large, cylindrical tissue sample. **(D)** Withdraw the rotary cut tube, expose the needle tip, and transfer tissue samples to a dedicated sample tray.

The information and pathological records of patients who underwent CassiII or Core needle biopsy and subsequent SE at our institution between January 2019 to December 2021 were retrospectively collected and analyzed. Clinicopathologic data of age; tumor location; histologic type; grade; pTNM category; immunohistochemical staining results of ER, PgR, HER2, and Ki67 were obtained through medical records and pathologic reports. CNBs used core needles were mostly performed by ultrasound guidance with 14-gauge needles and 3–5 pieces were received from mass forming lesions. The CassiII needle biopsy were performed by ultrasound guidance as well but with 10-gauge needles and 2-3 pieces were obtained for pathologic examination. The different steps in the two biopsy procedures can be seen in **Figure 1**. All biopsy and surgery specimens were processed and diagnosed according to national guidelines (17, 18).

The subjective and objective information of patients between July 2021 and March 2022 were recorded on the next day after CassiII biopsy in order to understand the complications. Coagulation function, pain, post-puncture compression and ecchymosis were recorded.

### 2.2 Pathology

Formalin-fixed, paraffin-embedded biopsy and surgery specimens were obtained for histopathologic analysis. Briefly, biopsy specimens were fixed in 10% neutral formalin for 8–11 hours, embedded in paraffin, and sectioned at 4-μm thickness. Hematoxylin and eosin (H&E) staining was performed subsequently. Surgical specimens were cut into 0.5–1 cm thick section after the operation and fixed in 10% neutral formalin for 8–24 hours; H&E staining was subsequently performed. IHC was performed by immunohistochemistry autostainer (Ventana Benchmark Ultra and Dako AS Link 48). The primary antibodies applied were antiestrogen receptor (ER, clone SP1; Roche), antiprogesterone receptor (PgR, clone 1E2; Roche), anti-Ki67 (clone MIB-1; Dako), and anti-Her2 (clone 4B5; Roche). ER status, PgR status, Ki67 labeling index, and Her2 were evaluated for each specimen.

For ER and PgR status the proportion of positive-stained tumor cells (the proportion score) were considered as follows: 0, no cells stained positive; 1, 0%–1% positive; 2, 1%–10% positive; 3, 10%–33% positive; 4, 33%–66% positive; and 5, 66%–100% positive. Moreover, two categories (negative/positive) were rated according to pre-determined cut-off values (1% for ER and 20% for PgR) based on values commonly used in the scientific community.

HER2 status assessed by IHC was categorized as follows: 0, no staining or membrane staining which faint, incomplete, or barely perceptible in less than 10% of tumor cells; 1+, incomplete membrane staining in more than 10% of tumor cells that was faint or barely perceptible; 2+, weak to moderate membrane staining (considered equivocal) in more than 10% of tumor cells; and 3+, intense and complete membrane staining in more than 10% of cancer cells.

In stage I or II breast cancer patient group, the International Ki67 in Breast Cancer Working Group (IKWG) builds a consensus that Ki67 30% or more, can be applied to estimate prognosis. Thereby, the cutoff value for Ki67 was 30%. The standard Ki67 protocol incorporated the updated recommendations of the IKWG (19).

### 2.3 Activated partial thromboplastin time

Blood left over after clinical testing was deposited into a sodium citrate anticoagulated tube (2.7 mL) containing 0.5 mL sodium citrate (0.109 mol/L). The citrate anticoagulated sample was centrifuged for 5 minutes at 3000 rpm. The citrated plasma was separated aseptically and assayed using manual methods for APTT using Diagen Diagnostics reagent (Diagen Reagents Ltd, Thame, UK). Test procedures were conducted according to the standard instructions. The plasma samples were cooled and rewarmed and the APTT assay was performed. The APTT was determined at the following temperatures: 40°C, 38°C, 37°C, 36°C, 34°C, 32°C, 30°C, 25°C, 20°C, 15°C and 10°C. Then the sample was frozen and rewarmed immediately and the APTT was determined at the following temperatures: 10°C, 20°C, 30°C, 34°C, 37°C and 40°C.

### 2.4 Statistical analysis

The histologic type and CRs of receptor status between biopsy and histology samples were calculated as percentage and Kappa value. K-values <0.20 were defined as poor concordance, 0.21–0.40 fair agreement, 0.41–0.60 moderate agreement, 0.61–0.80 good agreement, and 0.81–1.00 very good agreement. The SE pathological diagnosis was considered the gold standard while the biopsy histology was the test assessed when calculating sensitivity, specificity, positive and negative predictive values, pathological underestimation rate and overall accuracy. Counting data were analysed using the chi-square test or Fisher's exact test. For continuous variables satisfying normal distribution, they were expressed as mean ± standard deviation (mean ± sd), and differences between groups were tested by independent t-test. For measurement data that did not satisfy normal distribution, they were expressed as median (M) [interquartile range (IQR)], and differences between groups were analysed using the Mann-Whitney U test or the Kruskal-Wallis test. P-values of < 0.05 were considered significant. All statistical tests were performed using the SPSS statistical software package (version 22.0; SPSS Company, Chicago, IL). Sankey diagrams depicting changes in proportion score were computed in Sankey MATIC (http://sankeymatic.com)

## 3 Results

### 3.1 Patient characteristics

**Figure 2:**
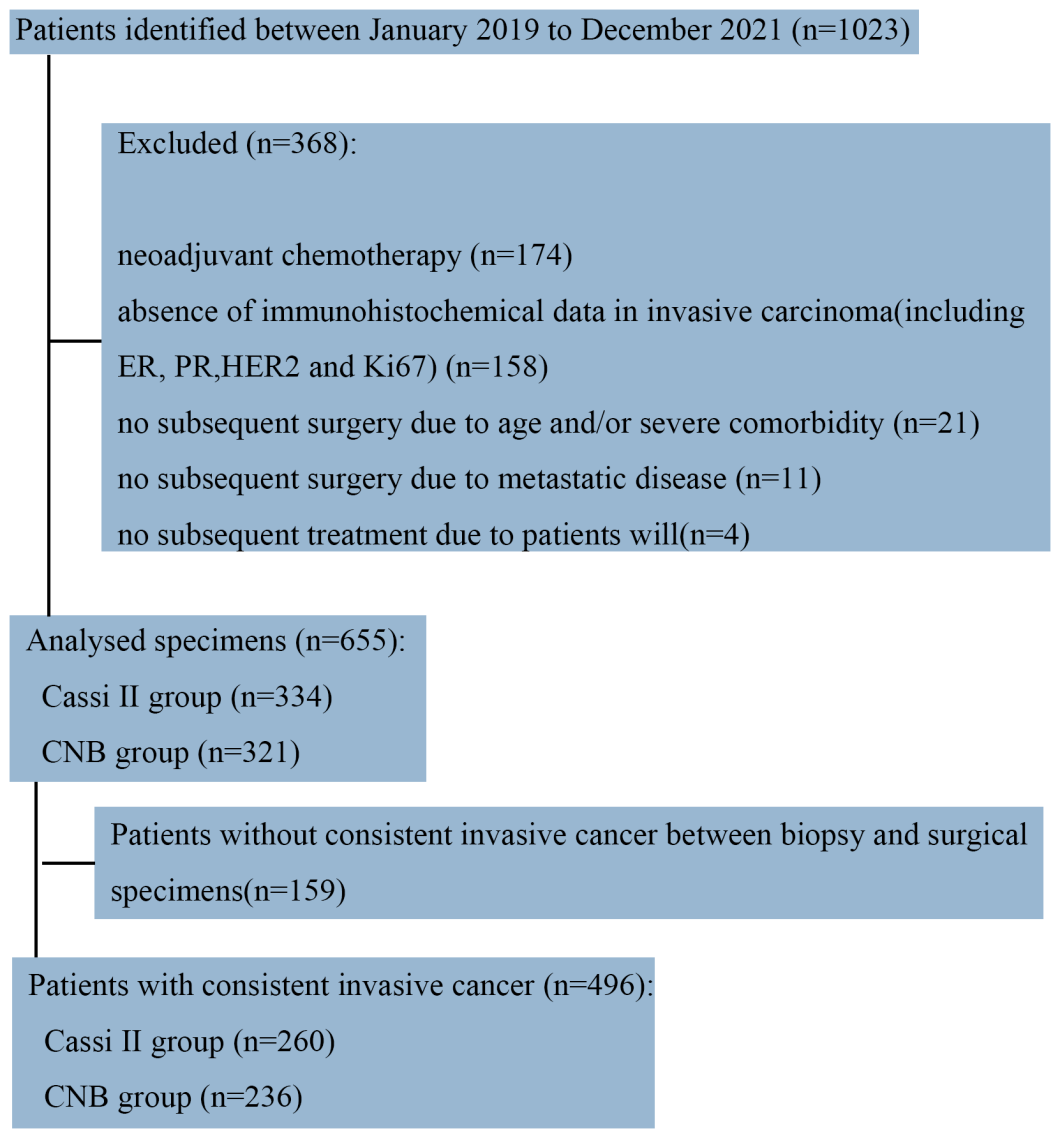
Flowchart of patient selection from Sir Run Run Shaw Hospital.

To compare CassiII needle biopsy and CNB, we retrospectively obtained data of the patients with breast cancer from the Department of Surgical Oncology, Sir Run Run Shaw Hospital from January 2019 to December 2021 (N=1023). A total of 174 patients who were treated with neoadjuvant chemotherapy or endocrine therapy were excluded to avoid the potential alteration of immunohistochemical parameters (particularly the proliferation rate) as a consequence of the treatment. Patients who had undergone no surgery and samples which were determined as invasive carcinoma by biopsy but without immunohistochemistry were excluded (**Figure 2**).

As a result, 655 samples were included in the present analysis, of which 334 cases received pre-operative Cassi puncture while the other 321 cases accepted Core needles biopsy before surgery. The median age of Cassi group and Core group was 57 and 56. The most common pathological tumor type in both groups was invasive carcinoma of no special type (75.14% vs. 78.50%). Demographics and pathology features between the two groups were shown in **Table 1**; **Supplement Table 1**.

**Table 1:** Patients and clinicopathological characteristics.

Variable		
	CassiII (N=334)	Core (N=321)
Age, yr [M, (IQR)]	57 (15)	56 (17)
Pathological type, number (%)		
Carcinoma in situ	41 (12.28)	43 (13.40)
Ductal carcinoma in situ	32 (9.58)	35 (10.90)
Intraductal papilloma	3 (0.90)	3 (0.93)
Solid papillary carcinoma	5 (1.50)	3 (0.93)
Apocrine ductal carcinoma in situ	1 (0.30)	1 (0.31)
Paget’s disease	0 (0.00)	1 (0.31)
Invasive carcinoma	293 (87.72)	278 (86.60)
Invasive carcinoma of no special type	251 (75.14)	252 (78.50)
Mucinous carcinoma	13 (3.89)	10 (3.12)
Invasive lobular carcinoma	9 (2.70)	3 (0.93)
Invasive micropapillary carcinoma	3 (0.90)	4 (1.25)
Neuroendocrine carcinoma	4 (1.20)	1 (0.31)
Minor pathological type	13 (3.89)	8 (2.49)
Pathologic T category, number (%)		
pTis	41 (12.28)	43 (13.39)
pT1mi	8 (2.39)	12 (3.43)
pT1a	8 (2.39)	14 (4.36)
pT1b	15 (4.49)	15 (4.67)
pT1c	152 (45.51)	121 (37.70)
pT2	105 (31.44)	113 (35.20)
pT3	5 (1.50)	4 (1.25)
Pathologic N category, number (%)		
pN0	244 (73.05)	228 (71.03)
pN1	64 (19.16)	59 (18.38)
pN2	19 (5.69)	22 (6.85)
pN3	7 (2.10)	12 (3.74)
M category, number (%)		
M0	333 (99.70)	320 (99.69)
M1	1 (0.30)	1 (0.31)

### 3.2 Pathological diagnosis concordance

We next analyzed the concordance of pathological type within malignant surgical specimens between CassiII and CNBs. The CRs of malignant surgical specimens with CassiII and CNBs for pathological diagnosis were 88.02% (kappa, 0.598; p <0.001) and 81.62% (kappa, 0.458; p<0.001) (**Table 2**). There was a statistically significant difference in CRs between Cassi group and Core group (p =0.018). Precisely, accuracy of carcinoma *in situ* with Cassi was 82.93% while that with CNBs was 60.74%, revealing the better ability to distinguish carcinoma *in situ* from invasive carcinoma with CassiII (p =0.023). Positive predictive value of carcinoma *in situ* with Cassi was 64.15% and that with CNBs was 50.98% as shown in **Supplement Table 2**.

**Table 2:** Concordance of pathological type within malignant surgical specimens between CassiII and CNB.

Surgical specimen	CassiII (N=334)			Concordance rate (%)	Kappa	P value	Core (N=321)			Concordance rate (%)	Kappa	P value
	benign lesion	carcinoma in situ	Invasive cancer				benign lesion	carcinoma in situ	Invasive cancer			
carcinoma in situ	7	34	0	88.02	0.598	<0.001	16	26	1	81.62^*^	0.458	<0.001
Invasive cancer	14	19	260				17	25	236			

^*^p value=0.018.

### 3.3 Analysis of risk factors for pathological inconsistencies

When each characteristic was examined separately, tumor sizes were proved to be the risk factors for both CassiII needle biopsy as well as CNBs (χ^2 = ^22.851; p <0.001 vs. χ^2 = ^44.586; p <0.001). It was demonstrated that there was no significant association between pathological inconsistencies and other variables with CassiII. Nevertheless, pathological characteristics including age and lymph node metastasis were identified the risk factors for pathological inconsistencies with CNBs (**Table 3**).

**Table 3:** Univariate regression analysis of risk factors for pathological inconsistencies with CassiII and Core.

Pathological characteristics	CassiII (N=334)		X^2^	p value	Core (N=321)		X^2^	p value
	Inconsistency	Consistency			Inconsistency	Consistency		
Age								
<50	9	70	0.033	0.855	28	63	12.995	<0.001
≥50	31	224			31	199		
Location								
upper inner quadrant	8	67	1.495	0.828	8	46	2.155	0.707
lower inner quadrant	2	20			5	23		
central region	1	13			2	13		
upper outer quadrant	26	163			37	138		
lower outer quadrant	3	31			7	42		
Tumor size								
<1.5cm	14	26	22.851	<0.001	32	38	44.586	<0.001
≥1.5cm	26	268			27	224		
Lymph node metastasis								
Neg.	34	210	3.294	0.070	51	177	8.345	0.004
Pos.	6	84			8	85		
ER positive proportion								
-	8	54	3.975	0.553	21	70	2.775	0.735
0-1%	1	8			0	3		
1%-10%	1	6			2	7		
10%-33%	3	7			1	7		
33%-66%	2	9			2	8		
66%-100%	25	210			33	167		
PgR positive proportion								
-	12	80	3.101	0.684	23	96	2.027	0.845
0-1%	2	12			1	11		
1%-10%	1	33			4	14		
10%-33%	3	24			7	30		
33%-66%	5	30			4	29		
66%-100%	17	115			21	82		
HER2 status								
-	7	59	0.969	0.809	11	39	1.351	0.717
1+	9	73			18	82		
2+	16	120			16	88		
3+	8	42			14	53		
Ki67 positive proportion								
<30%	35	242	0.669	0.413	52	207	2.575	0.109
≥30%	5	52			7	55		

A multivariable analysis was subsequently performed utilizing logistic regression in order to further investigate these significant associations above in core needle group. The tumor size and the age were confirmed to have a statistically significant correlation with pathological inconsistency (**Table 4**). Compared to patients less than 50 years old, patients who were 50 or greater had a lower risk of pathologic incompatibility (OR=0.369; 95%CI: 0.194-0.699; p=0.002). The risk of pathological mismatch was 5.901 times higher for tumors larger than 1.5cm than for tumors no larger than 1.5cm (95%CI: 3.102-11.225; p<0.001).

**Table 4:** Multivariate regression analysis of risk factors for pathological inconsistencies with CNB.

Pathological characteristics	OR	95% CI	P value
Age			
<50	1.000	–	–
≥50	0.369	0.194-0.699	0.002
Tumor size			
≥1.5cm	1.000	–	–
<1.5cm	5.901	3.102-11.225	<0.001
Lymph node metastasis			
Neg.	1.000	–	–
Pos.	0.445	0.193-1.027	0.058

### 3.4 Hormonal receptors, HER2 status, and Ki67 concordance

Agreement between CassiII and CNB with SE for ER, PgR, HER2 and Ki67 proliferative index results are summarized in **Table 5**.

**Table 5:** Concordance between CassiII/CNB and surgical specimen for ER, PR, HER2, and Ki67 results.

Surgical specimen	CassiII (N=260)				CRs	Kappa	p-value	Core (N=236)				CRs	Kappa	p-value
	Neg	Pos	Equi	Total (%)				Neg	Pos	Equi	Total (%)			
ER														
Neg	51	3		54 (20.77)	98.08	0.941	<0.001	60	6		66 (27.97)	96.61	0.914	<0.001
Pos	2	204		206 (79.23)				2	168		170 (72.03)			
Total	53 (20.38)	207 (79.62)						62 (26.27)	174 (73.71)					
PgR														
Neg	99	6		105 (40.38)	90.77	0.812	<0.001	105	5		110 (46.61)	89.41	0.789	<0.001
Pos	18	137		155 (59.62)				20	106		126 (53.39)			
Total	117 (45.00)	143 (55.00)						125 (52.97)	111 (47.03)					
HER2														
Neg	104	1	17	122 (46.92)	69.62	0.482	<0.001	84	0	25	103 (46.19)	72.88	0.573	<0.001
Pos	2	24	7	33 (12.69)				0	42	5	43 (19.92)			
Equi	49	3	53	105 (40.38)				25	9	46	78 (33.90)			
Total	155 (59.62)	28 (10.77)	77 (29.62)					109 (46.19)	51 (21.61)	76 (32.20)				
Ki67	<30%	≥30%						<30%	≥30%					
<30%	197	12		209 (80.38)	86.92	0.552	<0.001	156	27		183 (77.54)	79.66	0.439	<0.001
≥30%	22	29		51 (19.62)				21	32		53 (22.46)			
Total	219 (84.23)	41 (15.77)						177 (75.00)	59 (25.00)					

**Figure 3:**
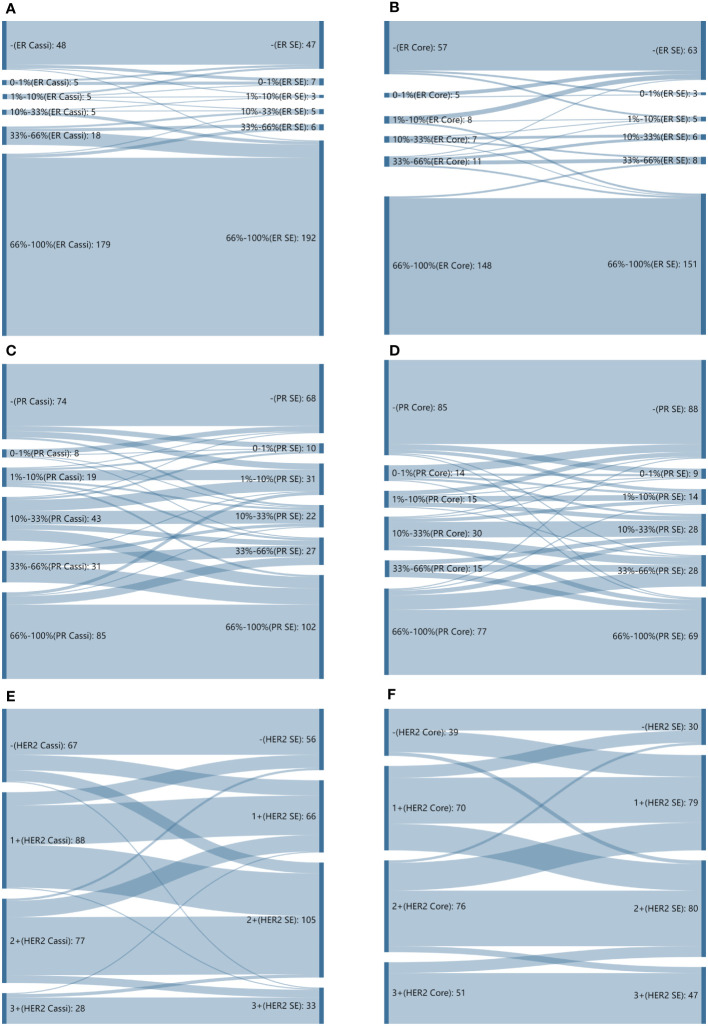
Sankey diagrams depicting changes in proportion scores. **(A)** Sankey diagrams depicting changes in proportion scores for estrogen receptor (ER) from CassiII needle biopsy to surgical specimen. **(B)** Sankey diagrams depicting changes in proportion scores for estrogen receptor (ER) from core needle biopsy (CNB) to surgical specimen. **(C)** Sankey diagrams depicting changes in proportion scores for progesterone receptor (PgR) from CassiII needle biopsy to surgical specimen. **(D)** Sankey diagrams depicting changes in proportion scores for progesterone receptor (PgR) from core needle biopsy (CNB) to surgical specimen. **(E)** Sankey diagrams depicting changes in proportion scores for HER2 from CassiII needle biopsy to surgical specimen. **(F)** Sankey diagrams depicting changes in proportion scores for HER2 from core needle biopsy (CNB) to surgical specimen. SE, surgical excision.

The CRs of surgical excision with CassiII and CNB for ER were 98.08% (kappa, 0.941; p<0.001) and 96.61% (kappa, 0.914; p<0.001), respectively. Concordance between the PgR assessment of CassiII and SE was found in 236 cases (90.77%; kappa, 0.812; p<0.001), with slightly higher than that in CNB (89.41%; kappa, 0.789; p<0.001). Changes in the proportion score for ER and PgR are shown in **Figure 3**. The CRs of surgical excision with CassiII and CNB for Ki67 were 86.92% (kappa, 0.552; p<0.001) and 79.66% (kappa, 0.439; p<0.001). The CRs of HER2 were 69.62% in CassiII group and 72.88% in CNB group, both with moderate agreement (kappa, 0.482 vs. 0.573; p<.001), which were lower than for hormonal receptors and Ki67 due to the equivocal status of IHC in both biopsy and surgical specimens. Changes in each grade for HER2 from CassiII or CNB to surgical specimen were shown in **Figure 3**.

### 3.5 The complications of CassiII needle biopsy

We collected and recorded 132 patients who received the CassiII needle biopsy in order to explore the potential complications. Each patient who underwent the biopsy was put under pressure for 30 minutes. Except for 4 patients who did not wear a compression corset for continuous compression, the rest of the patients all wore the corset, and 7 of 132 patients even utilized sandbags for pressure hemostasis. Six patients experienced chest tightness and discomfort after applying pressure with sandbags. Nearly half of all patients complained of mild pain lasting several hours after puncture, and 23 of 132 patients observed minor subcutaneous ecchymosis. (**Table 6**).

**Table 6:** Complications of CassiII needle biopsy.

Complications	No. (%)
Total number	132
Oppression way	
gauze	4 (3.03)
corset + gauze	121 (91.67)
sandbag + corset + gauze	7 (5.30)
Pain(NRS score)	
Neg.	68 (51.52)
score 1	40 (30.30)
score 2	22 (16.67)
score 3	2 (1.52)
Subcutaneous ecchymosis	
Neg.	23 (17.42)
Pos.	109 (82.58)
Chest stuffiness	
Neg.	126 (95.45)
Pos.	6 (4.55)

### 3.6 Effect of temperature on endogenous coagulation system

**Figure 4:**
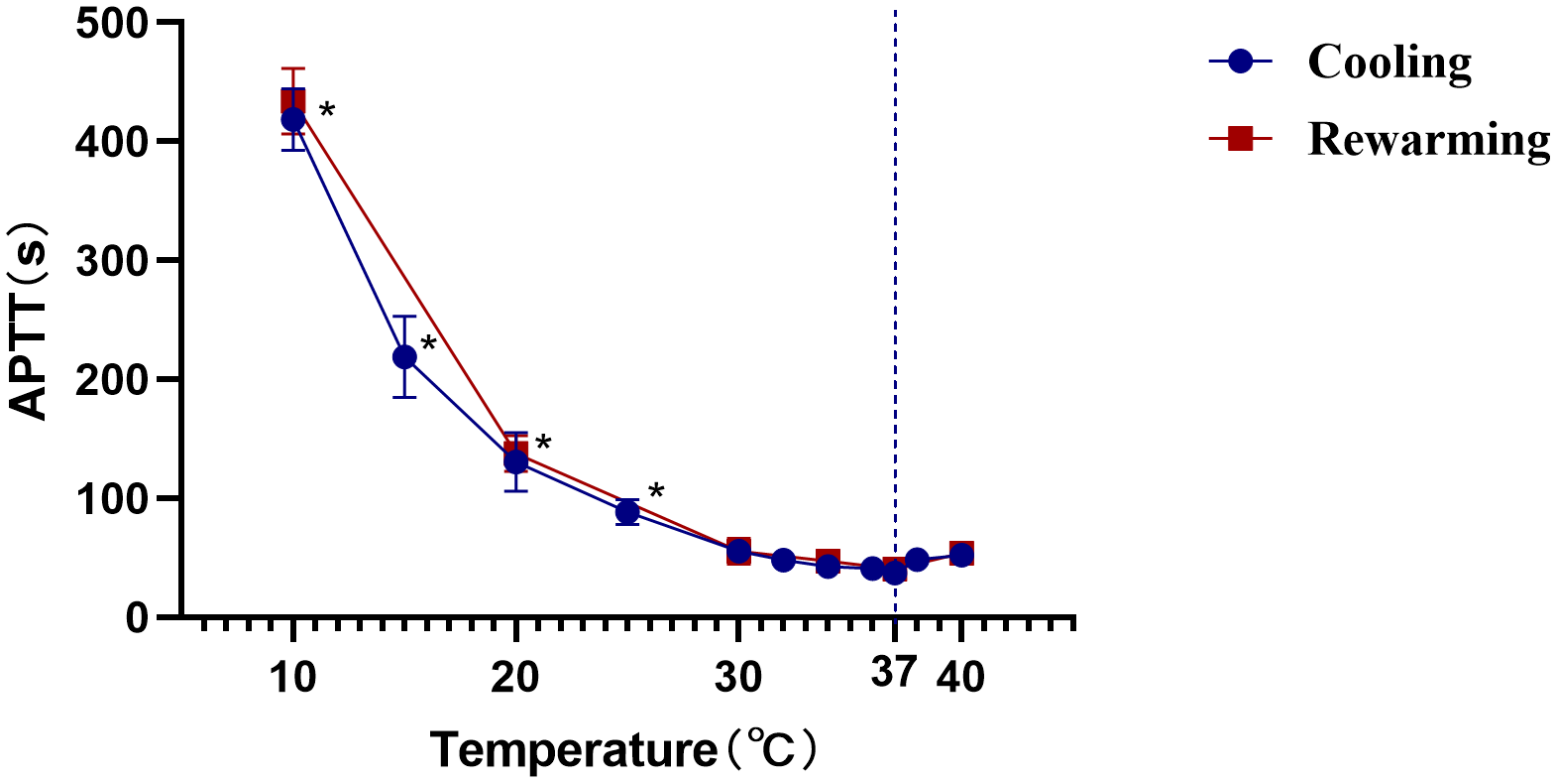
Effect of temperature on APTT clotting time. The APTT in seconds of normal pooled plasma (determined as described in the Materials and Methods section) is plotted against the temperature. ^*^, Statistically significantly different from that at 37°C at the p<0.05 level. The data shown are the mean and SD of three separate assays.

We determined the effect of temperature on a standard APTT assay. The clotting time of normal pooled plasma (NPP) at 37°C was the shortest with 37.48 seconds. The clotting time of NPP showed no significant difference between 30°C and 40°C, suggesting no net effect of temperature on overall plasma enzyme activity between these temperatures. However, the clotting times at 25°, 20°, and 10°C were significantly prolonged compared with 37°C (p <0.05, n=3) (**Figure 4**).

## 4 Discussion

Early detection and treatment of breast lesions greatly affect the prognosis of patients and living quality. US-guided needle biopsy has become an accepted technique for diagnosis of breast abnormalities, and it may help patients avoid the higher morbidity and cost of surgical excision. The results indicated that the tissue number of biopsies acquired from CNB affected the histopathological results and biomarker status (11, 20). The earlier study reported that it was more frequent for CNB to miss the components of invasive carcinoma because of the insufficient tissue ribbons, causing diagnosis underestimation. This phenomenon indicated that CNB had major deficiency in the diagnosis of invasive carcinoma and carcinoma *in situ* for breast lesion (21). Huo et al. reported that approximately 20% of ductal carcinomas *in situ* (DCIS) declared by CNB were proved to be invasive carcinoma according to postoperative diagnosis (22). The larger needle size of the CassiII needle allows enough tissue to be obtained and may improve the accuracy of pathological diagnosis. To our knowledge, it is the first article which has compared the accuracy and consistency of pathological diagnosis and biomarker status between CassiII and core needle biopsy.

In this study, we found that CassiII needle biopsy had good accuracy and consistency in diagnosing and distinguishing carcinoma *in situ* and invasive carcinoma. Compared with CNB, CassiII needle biopsy had a higher CR (88.02% vs. 81.62%, p=0.018) with moderate agreement. There was a significant difference in sensitivity of carcinoma *in situ* between the two biopsy methods which revealed that CassiII needle biopsy had a better ability to distinguish carcinoma *in situ* from invasive carcinoma than CNB (82.93% vs. 60.47%, p=0.023). Previous studies have indicated that the accuracy was lower for diagnosis of DCIS than for invasive cancer with CNB (23–25). Additionally, when a diagnosis of pure DCIS is made by CNB, but an associated invasive component is discovered in the surgical specimen, raising the possibility of lymph node metastasis (26). Therefore, it is significant to figure out whether there are invasive components in the tumor before surgery for patients to choose the surgical method (27). Comparative analysis of the underestimation rate of invasive cancer demonstrated that CassiII needle biopsy had a lower underestimation rate than CNB, mainly because CassiII needle could obtain sufficient specimens during biopsy and provide more histological information. In fact, CassiII is obviously more expensive than the CNB and this excess cost is to be paid by the patients. However, accurate preoperative identification of suspicious breast lesions is important in guiding surgical planning, reducing the number of rapid intraoperative pathologies, shortening the duration of the subsequent operation, and reducing anesthesia time.

The clinical and pathological characteristics affecting the pathological consistency of the two methods were further analyzed. We found that the tumor size was the only clinical feature which impacted pathological consistency with the CassiII needle biopsy group while there were more influence factors in the CNB group involving age, tumor size and status of lymph node metastasis. Multivariate analysis in the CNB group revealed that age ≥50 years old (OR:0.396, p=0.002) and tumor size ≥1.5cm (OR:0.169, p<0.001) were protective factors for pathological consistency. Due to the limited amount of tissue obtained by biopsy needles it was easier to penetrate the tumor component for the larger mammary lump which was consistent with our knowledge. The early studies showed a significant association between breast density and age is evident. Increasing age was associated with decreasing breast density (28, 29). Core needles have ejection force during biopsy, so it is difficult to receive correct tissue ribbons in dense breast, which could explain the higher consistency of pathological diagnosis in patients who are >50 years old. In the process of CassiII needle biopsy, the biopsy site is frozen partly and then cut system rotates to ensure that the target tissue would not be deflected or moved forward, therefore, it is not affected by the density of the mammary. This result above indicated that it had a wider applicability with CassiII needle as the consequence of the fewer restrictions on the clinical application. We found that lymph node metastasis status showed no significant difference in multivariate analysis and could be explained for the lymph node metastasis status was a confounding bias. Early studies reported that a positive relation was found between the percent of cases with lymph node metastasis and tumor diameter (30–32). Larger tumor size increases the likelihood of lymph node metastasis in invasive breast cancer.

Accurate assessment of estrogen receptor (ER), progesterone receptor (PgR), human epidermal growth factor receptor-2 (HER2) status and Ki-67 proliferative index were the key-point to tailor invasive breast cancer treatment. These biomarkers are also used to predict therapy response and long-term prognosis (33, 34). The CRs of ER, PgR, HER2, and Ki67 status in CassiII needle biopsy and CNB specimens prior to surgical operation and those biomarkers from surgical specimens were analyzed in order to confirm the accuracy of molecular classifications performed by two biopsies. ER status showed both high CRs of 98.08% (kappa, 0.941) and 96.88% (kappa, 0.921) in two groups, and the CRs of PgR status were also high with 90.77% in CassiII group (kappa, 0.812) and 89.73% in Core group (kappa, 0.796), respectively. Nevertheless, a greater distribution and bigger change of proportion score from both two biopsy specimens to surgical excisions were noted for PgR than ER (**Figures 3A, B**). These findings above are consistent with previous studies which may be due to the reason that PgR is more heterogeneously distributed (26, 35, 36). Identifying HER2-positive breast malignant tumor patients prior to surgery is important since neoadjuvant or postoperative adjuvant HER2-targeted therapy is an effective option for these patients. In this study, we found that CNB had a higher concordance rate and greater distribution and bigger change of HER2 status than that of CassiII needle biopsy (72.88% vs. 69.62%). A previous meta-analysis reported that the specificity of HER2 detection in CNB would be improved with a very low false positive rate (specificity 98%) with the HER2 positivity definition as IHC 3+ or FISH+ (37). The absence of FISH resulted in a significant decrease in the concordance rates of two biopsy groups in the report. Ki-67 proliferative index has been a widely used cell proliferation marker in cancer for several decades, and a meta-analysis has shown that high Ki67 expression impacts a greater risk for distant relapse and a worse outcome (38). There was an increasing controversy about the standard cutoff value for Ki67 high expression and the lack of standardization of Ki67 pathological interpretation. A previous study showed that the CR for Ki67 between CNB and surgical specimen was associated tightly with the increased number of needles passes because of the tumor heterogeneity; however, a plateau was reached after more than six core passes (39). In the current study, Ki67 acquired by CassiII needle revealed better concordance with surgical specimen Ki67 which can be explained by the more tissue get from CassiII needle (CR, 86.92% vs. 79.66%, p=0.03; kappa, 0.552 vs. 0.439, respectively). It could be found that CassiII had a higher consistency in hormonal receptors and Ki67, and the limitation of the patients may be the reason for the lack of significant difference.

Finally, we explored the complication rates after the CassiII needle operation for a comprehensive evaluation. In the early review, the complication rate in CNB was 0.39%, including bleeding, hematoma formation and pain (40). In the current study, we reported that the complication rate of subcutaneous ecchymosis in CassiII needle biopsy was 17.42% and patients acquired CassiII needle biopsy described the slight pain. The significantly increased incidence of subcutaneous ecchymosis was associated with the larger diameter of the CassiII needle and the tissue transient freezing technique. Many studies showed that temperature had a significant impact on coagulation function due to the large number of enzymatic reactions in the coagulation process (41, 42). Because prothrombin time was too short to be accurate, only the influence of temperature on ATPP was evaluated in this study. As an enzymatic reaction, the APTT had the shortest time at 37°C and the same trend was observed in the plasma rewarmed after freezing. The transient freezing of tissue by CassiII needle partly affected blood clotting time, explaining the higher incidence of subcutaneous congestion after CassiII needle biopsy. Therefore, compared with CNB, adequate compression after CassiII needle biopsy is essential, which can effectively avoid bleeding or hematoma after operation.

## 5 Conclusion

The CassiII device has larger diameter (10-gauge: 3.4 mm) than core needle (14-gauge: 2.1 mm) and immobilizes the lesion by using Stick-Freeze Technology™ which secures the targeted tissue to the needle. CassiII needle biopsy is age-independent technique which has a better sensitivity than CNB for distinguishing carcinoma *in situ* and invasive carcinoma. The underlying mechanism of subcutaneous congestion or hematoma after CassiII needle biopsy might be the larger needle diameter and the effect of temperature on coagulation function. Adequate compression can effectively avoid the occurrence of serious complications after the CassiII needle biopsy.

## Data availability statement

The raw data supporting the conclusions of this article will be made available by the authors, without undue reservation.

## Ethics statement

The studies involving human participants were reviewed and approved by Sir Run Run Shaw Hospital, Zhejiang University School of Medicine. Written informed consent for participation was not required for this study in accordance with the national legislation and the institutional requirements.

## Author contributions

SJ, SX, and LW designed the study, and SJ, SX, JHZ, and XZ collected the information. SJ, XZ, and JHZ analysed the data. SJ and CQ performed the experiments. SJ and SX contributed to writing the manuscript. YC, MM, JCZ, FJ, and LW contributed to manuscript review and revision. All authors contributed to the article and approved the submitted version.
