# Supplementary material for: A retrospective comparative study on the diagnostic efficacy and the complications: between CassiII rotational core biopsy and core needle biopsy
Source: Front Oncol. 2023 Sep 26;13:1067246. doi: 10.3389/fonc.2023.1067246 (PMC10562690; doi:10.3389/fonc.2023.1067246)
Supplement: Supplementary file 1 [file Table_1.docx]

Supplementary Material

**Supplement Table 1.** Minor pathological type from Table 1

| Pathological type | No. (%) | |
| --- | --- | --- |
|  | CassiII | Core |
| Invasive papillary carcinoma | 4 (1.19) | 2 (0.62) |
| Tubular carcinoma | 1 (0.30) | - |
| Mixed invasive ductal and lobular carcinoma | 1 (0.30) | 1 (0.31) |
| Mixed invasive ductal and micropapillary carcinoma | - | 1 (0.31) |
| Apocrine carcinoma | 3 (0.90) | 3 (0.93) |
| Carcinoma with medullary feature | 1 (0.30) | - |
| Metaplastic carcinoma | 3 (0.90) | 1 (0.32) |

**Supplement Table 2.** Diagnostic efficiency of CassiII and CNB for evaluation of pathological type in breast lesion

| Measure | CassiII (%) [95% CI] | Core (%) [95% CI] |
| --- | --- | --- |
| Accuracy of carcinoma in situ | 82.93 (34/41) [70.90-94.95] ^*^ | 60.47 (26/43) [45.24-75.69] |
| Accuracy of invasive cancer | 88.74 (260/293) [85.10-92.38] | 84.89 (236/278) [80.66-89.13] |
| Positive predictive value of carcinoma in situ | 64.15 (34/53) [50.81-77.50] | 50.98 (26/51) [36.78-65.18] |
| Positive predictive value of invasive cancer | 100.00 (263/263) | 99.58 (236/237) [98.75-100] |
| Pathological underestimation rate | 6.20 (21/456) [3.67-8.90] | 10.25 (33/289) [6.92-13.58] |
| Overall accuracy | 88.13 (297/337) [84.66-91.60] | 81.68 (263/322) [77.43-85.93] |

^*^, p value=0.023
